# There are considerable drawbacks to oral anticoagulant for monitoring patients at home which should lead family physicians to discuss alternative or enhanced solutions: a cross-sectional study

**DOI:** 10.1186/1471-2261-13-71

**Published:** 2013-09-11

**Authors:** Jean Yves Le Reste, Benoit Chiron, Bernard Le Floch, Patrice Nabbe, Marie Barrais, Jacques Mansourati, Sébastien Cadier, Pierre Barraine, Claire Lietard

**Affiliations:** 1 Département de médecine générale - UFR Brest, Rue Camille Desmoulins, 29200 Brest, France; 2 Département de cardiologie - CHU Brest la cavale Blanche, 29200 Brest, France; 3 Département de santé Publique – CHU Brest la cavale blanche, 29200 Brest, France

## Abstract

**Background:**

INR (International Normalized Ratio) is the biological reference test for the monitoring of vitamin K antagonist (VKA) therapy. Overdosage of VKAs causes about 17,000 hospitalizations and 5,000 deaths each year in France. To avoid these complications, monitoring and blood sampling conditions must be rigorous. In France, more than half of INRs are carried out at home. The aim was to determine blood-sampling conditions at home, transit time and the quality of the laboratory reagents used.

**Method:**

Questionnaire-based, descriptive epidemiological cross-sectional prevalence study involving home care nurses, family physicians (FPs) and clinical laboratories. Setting: Brittany, France, 2008. Study of the pre-analytical phase of INRs sampled at home and its influence on INR results.

**Results:**

The study included 291 FPs, 249 home care nurses, and 49 laboratories. 32.5% of reported INRs were outside the therapeutic range. Samples were drawn into unsuitable tubes in 5.5% of cases and delivered in a chilled condition in 9% of cases. In urban areas 50% of the tubes took more than 2 hours to reach the laboratory compared with 71% from rural areas. The average International Sensitivity Index (ISI) of the thromboplastin was 1.62. The INRs provided by the laboratories were not analyzable in 64.7% of cases where blood samples had been taken at home.

**Conclusion:**

Blood sample quality, transit time and the reagents used are currently inadequate. The majority of INRs taken at home are not reliable. FPs should consider these drawbacks in comparison with alternative solutions to increase patient safety.

## Background

Vitamin K antagonists have been taken orally for more than 40 years to ensure anticoagulation. Their main indications are venous thromboembolism, artificial heart valves and atrial fibrillation. The increasing prevalence of cardiovascular illnesses in an aging population across Europe and all developed countries means that the number of patients continues to increase [1] with about 900,000 patients currently receiving VKAs in France alone [2].

Even where results show benefits for the patient, the risk of hemorrhage and stroke remains an important consideration. In 2007, VKAs were responsible for 17,000 hospitalizations and between 4,000 and 5,000 deaths [3] in France. In the DREES [2] (hospital-based) study, just over 30% of serious, adverse outcomes linked to medication, were due to an anticoagulant. Irregularity in the supply of vitamin K causes an imprecise balance in the anticoagulation provided. Frequent blood testing is therefore essential, bearing in mind the risk of under- or over-dosage which could lead to thrombosis or bleeding.

When VKAs were first used, the monitoring was conducted using Prothrombin Time (PT) measurement, that is, the coagulation time for citrated blood plasma when in contact with a reagent known as calcic thromboplastin. Thromboplastin is a tissue factor which accelerates the process of coagulation. The use of PT in the biological monitoring of treatment has been replaced by the INR (International Normalized Ratio). This shows the ratio (patient PT/control PT) raised to the ISI power (International Sensitivity Index). The ISI reflects the sensitivity of the thromboplastin to the reduction in vitamin K dependent factors. In this mathematical formula, with the ISI being the exponent, the higher the thromboplastin ISI level, the greater the likelihood of mistakes in determining the PT and the higher the rate of error in calculating the INR.

Furthermore, the PT value is influenced by blood-sampling conditions and it is essential that certain sampling conditions are observed. The blood must be taken cleanly*,* preferably without a tourniquet, and collected in a tube containing citrate [4,5]. The tube must be reasonably full with, ideally, 9 parts blood to 1 part citrate. It must be turned upright smoothly without shaking the contents. Each tube must be subjected to centrifugation. Ideally, this should take place immediately after the blood has been drawn; it can then be stored for up to 4 hours before analysis. The centrifugation may be postponed for up to 2 hours, after which the analysis should be performed rapidly. Finally, the sample must be stored at an ambient temperature prior to analysis and must, under no circumstances, be chilled.

All these so-called pre-analytical conditions are normally well understood by analysis laboratories and that is why patients are recommended, wherever possible, to have their blood drawn at the laboratory. The INRs drawn at home represent 48% to 66% of all blood samples taken in France [6]. In daily practice, INR follow-up care is undertaken by Family Physicians (FPs). Other procedures, such as blood-drawing at a Family Physician’s office or self-measuring are not available in France, nor in many other European countries.

In 2006, for a thesis on family practice [7], a survey was conducted amongst FPs, biologists and home care nurses in one county in Brittany, on the practices involved in administering VKAs. This was the first French study of INR sampling conditions, at home, by home care nurses. The survey shows numerous incidences of incorrect tubes being used or samples being stored in chilled conditions. In addition, the transit time generally exceeded 2 hours, particularly in rural but also in urban areas. Finally, the thromboplastin used by the laboratories, had ISI values which were too high. These results led to doubts about the reliability of the INR obtained. The relatively restricted sample of professionals questioned and the limited geographic zone did not allow generalization.

The aim of this work was to identify INR sampling conditions at home, in Brittany, in order to gauge the reliability of the INR results obtained.

## Method

This study is a descriptive cross-sectional epidemiological prevalence study, to evaluate the practices of FPs, home care nurses and laboratory biologists using a specific questionnaire for each profession. The distribution and collection of the questionnaires took place between January and March 2008 in the four counties of Brittany. All FPs and home care nurses from Brittany were selected from the ADELI directories of the Regional Directorate for Health and Social Work covering urban and rural areas. Exclusion criteria for FPs: those whose practiced homeopathy, acupuncture or osteopathy as their main activity. For nurses, there were no exclusion criteria. Systematic randomization was undertaken, selecting 1 in every 3 FPs and 1 in every 5 nurses. All the medical analysis laboratories in those geographical areas were included in the study.

### Questionnaires

Each of the categories of professional received a questionnaire adapted to his/her practice (see Additional file 1). The first part concerned the socio-professional context and the second covered the practices relating to the biological monitoring of the VKA treatment.

One of the questions for the FPs and the nurses distinguished between types of practice environment: rural or urban. This feature enabled the differentiation of transit time according to practice environment.

All questionnaires had been tested in a pilot study. That study was undertaken to ensure there were no misunderstandings and no reversals of meaning due to sentence-structure.

The questionnaires were anonymous. They were sent by post with a letter explaining the study and a pre-paid envelope for their return. Participation was completely voluntary in every case.

Even though there was no patient involvement, the promoters of the study asked for an Ethical Committee Agreement. The Ethical Committee of the “Université de Bretagne Occidentale” (Brest, France) accepted the project.

## Results

The response rates were upper than 30% for every population and is described in Table 1.

**Table 1:** Response rate

**Recipients**		**FPs**	**Nurses**	**Biologists**
Questionnaires	Distributed	851	749	93
	Returned	294	249	49
	Completed	291	249	49
Response rate		33.8%	32.4%	52.7%

All response rates were upper than 30%.

The observed target value for the whole study are described in Table 2.

**Table 2:** INR observed target values

**Total INRs recorded**	**INR rate 2-3**	**INR rate <2**	**INR rate >3**	**INR 3–4.5**	**INR rate outside therapeutic range**
1224	58.5%	15.7%	25.8%	9%	32.5% (estimated)

More than one third of values were outside the therapeutic range.

### Nurse blood sampling conditions

5.5% of the nurses stated that they used non-citrate tubes (heparinized or EDTA) and 9% transported the tubes in chilled containers.

### Nurse blood sampling and transit times

The number of nurses whose stated practices did not conform to the recommendations was 64.7% (non-citrate tubes, transportation in chilled containers and transit times of > 2 hours). All transit times for both urban and rural practices are listed in Table 3.

**Table 3:** Transit times*

**District nurses**	**Number in survey**	**Average transit time for samples in minutes**	**Samples with a transit time >120 minutes**
Urban practice	169	110 ± 55	51%
Rural practice	80	181 ± 68	71%

(* Time necessary to transport the sample from home to laboratory).

51% of samples in urban practices and 71% in rural practices were delivered with a non-acceptable transit time.

### ISI values

In the laboratories, the ISI of the thromboplastins used to calculate the INRs had an average value of 1.62 ± 0.21; CI 95 = 1.21-2.02.

### Quality control

All the laboratories which responded stated that they ran daily in-house tests to check the variability of the results and 15% stated that they never ran external tests (i.e. between laboratories).

## Discussion

### Summary of main findings

The study of INR sampling conditions, at home, by nurses has shown the use of tubes which were inappropriate in certain instances (which is a major failing); transportation in chilled condition in other instances (which is an important failing) and analysis outside the time limit in the majority of cases (which is another important failing). These problems affected around 2 out of 3 samples taken.

As expected, the transit time results show a clear difference between urban and rural areas. However, the pooling of resources between laboratories is widespread in large urban centers, with samples deposited at the laboratory and only later sent for specialized technical analysis. This lengthens the actual transit time and reduces the rural/urban difference observed in this study.

Furthermore, the biologists who responded used thromboplastins which had an average ISI value of 1.62. During the calculation of the INR, these values increase the misleading PT variations from the pre-analytical stages (which is an important failing). Can we trust INR results from samples taken at home? The actual consequences of the gap between the INR obtained and the INR normally expected could be analyzed in studies that modify each of the individual parameters. Studies such as these exist [8,9] but they used high quality thromboplastins, with an ISI close to 1, which do not increase the errors in the pre-analytical phase. Other studies would, of necessity, have to be undertaken with higher ISI values.

The ‘external’ quality control tests compared the INR results for the same plasma between different laboratories. The variability obtained is usually between 12% and 15% in optimal, pre-analytical conditions. As the current study has found pre-analytical conditions that fall far short of the required standard, tests such as these, with samples taken at home, should logically show greater variability. Surely the iatrogenic effects, due to VKA, are at least partially linked to the problem raised in this study of sampling practices.

### Comparison with existing literature

As a comparison, the team undertook a search of existing research literature. They found that research papers have mainly compared ideal pre-analytical sampling conditions in primary care clinics, in INR clinics, as well as the use of self-assessment and computerized decision-support systems, with conditions in university hospital laboratories [10,11]. From these comparisons it has been concluded that INR results from any of these sources are safe and reliable. However, to our knowledge, no other pragmatic study has been undertaken which is able to show that INRs, sampled under current health-care conditions, at some distance from a laboratory, were either safe or reliable.

### Questions and considerations for clinical practice and future research

Health authorities have already disseminated information in campaigns to remind professionals of good practice in dealing with VKAs [12]. New information that is better targeted and better disseminated might avoid mistakes with the tubes or with the conditions of transportation. However, the problem of transit time remains. With their current level of pay and workload, would it really be reasonable to insist that nurses make a return journey to the laboratory after each sample has been taken, especially in rural areas? One possible solution would be to insist that laboratories employ more couriers, with the aim of delivering each sample within a maximum of 90 minutes, allowing the laboratories to receive and to process the sample within the time limit required. Making this available would incur additional costs for the laboratory which they might have to recover through their INR invoicing. This seems unlikely in the current economic climate of budget restrictions.

Another possibility would be to encourage biologists to use thromboplastins which have a lower ISI and values of around 1. In this way, errors in the PT calculation would not be increased when calculating the INR. However, certain technical constraints which hinder this solution need to be clarified. An ISI close to 1 is not ideal at the start of treatment, when the prothrombin level is still raised, as the INR does not reflect the degree of anticoagulation effectively. Currently, it is still recommended that biologists use thromboplastins with an ISI close to 1.5. However, these recommendations do not take into consideration the results of this study on the pre-analytical errors which occurred when samples were taken at home. In addition, the choice of thromboplastin and its ISI depends on the technical features of the equipment used in the laboratory. The biologist is therefore dependent on the manufacturer of the technical equipment, which often commercializes the use of a particular thromboplastin; consequently, this is another unsuitable alternative.

Possible alternatives present themselves with the recent appearance of new measuring devices [13] and new oral anticoagulants [14].

The new measuring devices could be used at home by the patient (if he is able) or be monitored by a nurse or an FP. However the time allocation for nurses or FPs would remain at a high level [15]. In addition, in many countries the laboratories would take industrial action to protest against loss of employment.

The new oral anticoagulant, belonging to the class of non-peptide thrombin inhibitors, heralds a new era in anticoagulation: one which will no longer require biological monitoring. Trials are under way to validate these products in the treatment of atrial fibrillation [14,16] and thromboembolism [17]. Early findings of these non-inferiority trials indicated a potential increase in patient safety using these new anticoagulants (compared with warfarin) [16] whilst, at the same time, being potentially more effective for stroke prevention in atrial fibrillation. If these indications are confirmed, it is highly likely that FPs will be tempted to transfer rapidly to this new class of anticoagulant in order to increase patient safety.

### Strengths and limitations of the study

The 3 groups of professionals questioned were not representative of the body of professionals practicing in France. There is a selection bias in favor of rural areas. For example, the urban/rural ratio for FPs was 70:30 in this study (representative of Brittany) whereas it was 81:19 in mainland France in 2006.

A further example of selection bias is present for nurses. The geographical area studied is not representative of France as there is unequal provision of nurses in different regions. Brittany is situated in a well-equipped area compared with the national average. A study [18] using the same method took place in the Midi Pyrenees region in 2008. This region is ranked 4^th^ in metropolitan France, in terms of home care nursing provision and higher than Brittany. The average transit time limits were shorter than 36 minutes, whether in urban or rural areas. If the transit time limits are correlated effectively at this level of provision, the results could be applied to other French regions, however, there would, no doubt, be other factors to take into consideration.

This study is subject to the biases which are inherent in any survey by questionnaire.

Selection bias: The response level of approximately one half, in the case of the analysis laboratories, and more than one third, in the case of the nurses and FPs selected, is reasonably good for this type of survey. However, despite randomization, the professionals who had agreed to respond were self-selected, either by availability or through their interest in the subject. Consequently, it is probable that these professionals have greater knowledge in this field which, in turn, reduces the proportion of errors observed when compared with actual practice.

Statement bias: this concerns the self-evaluation of practices undertaken by the professionals who responded. This evaluation cannot be completely objective. Generally the stated practices tend to be idealized, masking errors to some extent, whether consciously or otherwise. In addition, the feeling of having one’s performance judged is never completely removed by anonymity.

## Conclusion

It is possible to generalize from our results and apply them to most rural areas in Europe and to all health systems where samples are subjected to a long transit time. INRs taken at home do not meet pre-analytical sampling requirements in over half of cases. Laboratories are using Thromboplastin with a high ISI which magnifies the pre-analytical errors. These dysfunctions offer plausible explanations for part of the iatrogenic effects linked to VKAs.

Any feasible improvements appear to be almost impossible within the current structure of the French health system where tests are, in the majority of cases, completed after long transit times and for the most delicate patients (those in care facilities, nursed at home or bedridden). It is possible to generalize and apply these findings to most rural areas in Europe in the current economic climate of budget restrictions.

An innovative option could be to supply portable self-measuring devices to be used at home, by the patient, if possible. As an alternative, where necessary, measurement could be undertaken by a nurse or an FP. Another innovative option would be to replace VKAs with one of the new classes of anticoagulant, which does not require a biological test, once the indication for the patient has been validated and the possible incompatibilities evaluated.

Whilst awaiting these developments, the first recommendation is that patients are encouraged to have their INR blood samples drawn at the laboratory. For patients who cannot travel, nurses should be advised to draw the INR samples at the end of their round, or ensure that the samples reach the laboratory rapidly. FPs should take all these drawbacks into account before making a decision.

## Competing interests

There is no competing interest regarding this study for any of its authors. The funding was provided solely out of public funds from the Department of General Practice at Brest University.

## Authors’ contributions

All named authors have seen and agreed with the submitted version of the paper. LRJY designed the study, collected data, and wrote the article. CB collected data and reviewed the article. NP designed the study and reviewed the article. LFB designed the study and reviewed the article. BM reviewed the article. MJ reviewed the article. CS collected data and reviewed the article. BP reviewed the article. LC designed the study and reviewed the article. All authors accepted this publication.

## Pre-publication history

The pre-publication history for this paper can be accessed here:

http://www.biomedcentral.com/1471-2261/13/71/prepub

## Supplementary Material

Additional file 1 Questionnaires for Family Physicians, Nurses and Biologists. Click here for file
