# Supplementary material for: There are considerable drawbacks to oral anticoagulant for monitoring patients at home which should lead family physicians to discuss alternative or enhanced solutions: a cross-sectional study
Source: BMC Cardiovasc Disord. 2013 Sep 11;13:71. doi: 10.1186/1471-2261-13-71 (PMC3846900; doi:10.1186/1471-2261-13-71)
Supplement: Additional file 1 — Questionnaires for Family Physicians, Nurses and Biologists. [file 1471-2261-13-71-S1.doc]

**Questionnaires for Family Physicians, Nurses and Biologists.**

**Questionnaire for Family Physicians**

I – Socio-professional environment

1. In which type of environment do you practice?

urban (>2000 inhabitants) rural (<2000 inhabitants)

2) How many years have you been practicing? ..............................................................

3) Approximately how many INR results do you deal with on a weekly basis ? ………………………………………………………………………………………………….

II - Description of practices

4) Indicate the values of some of your most recent INR results (maximum of 10)……………………………………………………………………………………………….…………………………………………………………………………………...

5) Which test do you consider suitable for managing VKA treatment?

PT  INR

6) With what frequency do you prescribe the INR test?

- When you start a course of VKA treatment? ………………………….…………………

- When the INR is stable within the therapeutic range? ….…………………………………

- When the INR has de-stabilized? …………………………………………………………….

7) In your absence, to whom to do delegate the task of managing the INR results?

your locum your colleague the home-care nurse

the patient himself his family the laboratory

other :

8) Do you use the INR record book?

yes, always yes, often yes, sometimes rarely never

III - Personal experiences (to help our understanding of care practices)

9) Do you systematically apply the INR values recommended in medical literature to every patient taking a VKA?

yes no don’t know

10) If not, for which reason(s) ?

out of concern for a patient based on his advanced years.

where the patient has a history of iatrogenic incidents when taking a VKA.

because of an unfortunate personal experience when a VKA had been prescribed (hemorrhage, thrombosis…)

because you were unaware of these values

because the INR is not a reliable test

11) Which iatrogenic incident(s) are you most concerned about in a patient taking a VKA ?

none thrombosis hemorrhage don’t know

12) Why? ..............................................................................................................

………………………………………………………………………………………………….

13) What was the last incident due to VKAs that you can recall in a patient?

ecchymosis gingivorrhagia epistaxis hematuria

gastrointestinal bleeding metrorrhagia cerebral hematoma

other :

14) Is it important for you to have the INR results on the day of the test ?

yes no don’t know

VI - Coordination with other health-care professionals

A - the medical analysis laboratory

15) Do you advise patients to have their INR tested at the same laboratory?

yes, always yes, often yes, sometimes no

16) If yes, why?

you have confidence in this laboratory to keep the results consistent

the lab biologist is also a doctor the presentation of results (diagrammatic layout…)

the assurance that you will get the results the same day

other

B - Home-care Nurses

17) Do you tend to advise the patient to have the blood test carried out…:

at the medical analysis laboratory ? at home by a home-care nurse?

you do not give any advice in this matter.

18) If the home-care nurse is drawing the blood, what is your conception of the time limit to be observed between the drawing of the blood and its arrival at the analysis laboratory?

<2 hours < 4 hours < 6 hours < 8 hours

don’t know

19) On the basis of this conception, do you tend towards using a nurse or someone else?

yes no don’t know

C - Transmission of the INR results

20) Where the INR is within the therapeutic range:

*- do you usually receive the results of the INR from :*

the patient a member of his family the laboratory

the home-care nurse your own phonecall no one

other:

- *within what time frame do you receive the INR result ?*

the same day the next day later

- *by which means are you informed of the result ?*

by telephone by fax by Internet by mail other :

21) Where the INR is outside the therapeutic range (<1.5 or >4.5) :

- *do you usually receive the INR result from :*

the patient a member of his family the laboratory

the home-care nurse your own phonecall no one

other:

*- within what time frame do you usually receive the INR result ?*

< 3 hours < 12 hours < 24 hours < 48 hours > 48 hours

*- by which means are you usually informed of this result ?*

by Internet by fax by telephone by mail other

D – the patient

22) When the INR is within the therapeutic range, do you usually contact the patient?

yes no don’t know

23) If yes, what do you do ?

you phone the patient yourself to tell him that everything is fine.

you call the home-care nurse so that she can let the patient know that everything is fine.

you call the laboratory so that they can let the patient know that everything is fine.

don’t know.

24) Where this is the start of the treatment or there is an imbalance in the VKA medication, how do you usually let the patient know the change of dose ?

you call the patient in order to speak to him personally by phone.

you do a home visit.

you call him to a consultation.

you contact a member of his family and leave a message.

you contact the home-care nurse so that she can contact the patient.

you contact the laboratory so that they can contact the patient.

other:

E - «Ideal health-care coordination »

25) What would be your idea of “ideal coordination” between health-care professionals involved in following up patients taking a VKA?

……………………………………………………………………………………………………………………………………………………………………………………………………..

**Questionnaire for home-care nurses**

I - Socio-professional environment

In which type of environment do you practice?



urban (>2000 inhabitants) rural (<2000 inhabitants)

2) How many years have you been practicing?.................................................

3) Approximately how many patients taking vitamin K antagonists (VKAs) have you been treating this week?.................................................................................................

II - Description of practices

A - Monitoring of VKA treatment

4) Which test do you consider suitable for managing VKA treatment?

 PT  INR

5) Do your patients have an INR record book?

yes, always yes, often yes, sometimes rarely never

B - Drawing the blood sample for the INR

6) At what time of day do you draw the INR blood sample?

………………………………………………………………………………………………

……………………………………………………………………………………………

7) Are a patient’s blood samples always taken at the same time of day or does this vary? ……………………………………………………………………………………

……………………………………………………………………………………………….

8) How are they stored prior to arriving at the laboratory ?

…………………………..…………………………………………………………………………………………………………………………………………………………………………………………………….

9) What is the average delay between the actual blood drawing and its arrival at the analysis laboratory? …………………………………………………………………………………

10) What type of tube do you use to draw blood for the INR test? (be specific)………………………………………………………………………………………………………………………………………………………………………………………..

III - Personal experiences (to help our understanding of care practices)

11) Do you think that the INR is a reliable test for monitoring patients taking VKAs ?

yes no don’t know

12) What types of iatrogenic incident(s) are you most concerned about in a patient taking a VKA ?

any hemorrhages thrombosis don’t know

other:

13) Why ?................................................................................................................

………………………………………………………………………………………………..

14) What was the last incident due to VKAs that you can recall in a patient?

ecchymosis gingivorrhagia epistaxis hematuria gastrointestinal bleeding metrorrhagia cerebral hematoma other :

IV - Coordination with other health-care professionals:

A - The medical analysis laboratory:

15) Do you always send the tubes to the same laboratory for INR analysis of the sample ?

yes no don’t know

16) If yes, for which reason(s) ?

you have confidence in this laboratory to keep the results consistent

the patient’s family doctor requests it the patient requests it

the presentation of results (diagrammatic layout…) the speed of the results service

other:

B - Transmission of the INR results

17) After taking a blood sample do you find out the result of the INR?

yes, always yes, often yes, sometimes rarely never

18) If yes, who most frequently informs you of the INR results ?

*- where the INR is within the therapeutic range:*

the patient a member of his family the laboratory

the patient’s family doctor you make a call yourself

other :

- *where the INR is outside the therapeutic range  (<1.5 or > 4.5):*

the patient a member of his family the laboratory

the patient’s family doctor you make a call yourself

other:

19) If yes, by which means are you usually informed ?

*- where the INR is within the therapeutic range :*

by telephone by fax by Internet by mail other

*- where the INR is outside the therapeutic range (<1.5 or >4.5):*

by telephone by fax by Internet by mail other

20) If yes, within what time frame do you usually receive the result ?

- *where the INR is within the therapeutic range :*

the same day the next day later

- *where the INR is outside the therapeutic range (<1.5 or > 4.5):*

< 3 hours < 12 hours < 24 hours < 48 hours >48 hours

C - The doctor and the patient

21) Do you usually know the value range, within which the INR should fall, for the medication to be balanced?

yes, always yes, often yes, sometimes rarely never

22) If yes, how are you informed of this?

by the patient by a member his family by the laboratory

the doctor contacts you to inform you

you contact the doctor yourself to find out

other :

23) If you know the INR result the same day as the blood is taken, what do you do ?

*- where the INR is within the therapeutic range :*

you yourself maintain the same dose, without telling the doctor.

you maintain the same dose and you call the doctor to tell him.

you call the doctor to pass on the INR information and he tells you the dose to administer.

the doctor contacts you, anyway, to tell you what action to take.

you are not involved.

other:

*- where the INR is outside the therapeutic range*

you adjust the dose yourself without telling the doctor.

you adjust the dose and your call the doctor to tell him.

you call the doctor to pass on the INR information and he tells you the dose to administer.

the doctor contacts you, anyway, to tell you what action to take.

you are not involved

other:

24) If you are not involved in the adjusting of the VKA dosage, how do you usually let the patient know the dose he should take?

you call the patient in order to speak to him personally by phone.

you do a home visit.

you call him to your office.

you contact a member of his family and leave a message.

you contact his doctor so that he can contact the patient.

you contact the laboratory so that they can contact the patient.

other

25) Does it happen that some doctors ask you to manage the INR results yourself and adjust the VKA dose when they are absent for a day or so?

yes, always yes, often yes, sometimes rarely never

D - “Ideal health-care coordination”

26) What would be your idea of “ideal coordination” between health-care professionals involved in following up patients taking a VKA?

………………………………………………………………………………………………...…………………………………………………………………………………………………...………………………………………………………………………………………………

**Questionnaire for the biologist**

I - Description of practices

A - The drawing of the blood sample

1) At what time of day is blood usually drawn for INR sampling?

………………………………………………………………………………………………….

2) Are the blood samples always taken at the same time from each patient or does this vary ?………………………………………………………………………...…………………………

………………………………………………………………………………...…………………

3) How is the INR blood sample stored until its analysis?

……………………………………………………………………………………………...……

4) What is the average delay between drawing the INR blood sample and its analysis?

……………………………………………………………………………………….…………

.

5) What type of tube do you use ? (Please specify)

…………………………………………………………………………………..………………

………………………………………………………………………………..…………………

B - The analysis

6) Which type of thromboplastin do you use (recombinant, bovine, human)?

……………………………………………………………………………..……………………

7) What is its ISI ? …………………………………………….…………………………

8) Do you regularly change your thromboplastin?

yes, often as little as possible no

9) If yes, for what reason(s) ?

………………………………………………………………………………………...…………

…………………………………………………………………………………….……………..

10) And if yes, do you inform the doctor by stating it on the INR results sheet?

yes no don’t know

11) What kind of equipment do you use ?

………………………………………………………………………………..…………………………………………………………………………………………………………….

12) Is the sensitivity of your thromboplastin calibrated on the equipment you use ?

yes no

13) If no, do you recalculate the ISI ?



yes no

II - Personal experiences (to help our understanding of care practices)

14) Do you run quality control checks for the INR ?

within your own laboratory (internal checks) ? yes no

between different laboratories (external checks)? yes no

15) If yes, are they checks which:

use normal samples ? yes no

use abnormal samples? yes no

16) If yes, how frequently?

- in the case of internal checks?.................................................................………....

…………………………………………………………………………………………………...

- in the case of external checks?…………………………………………………….

…………………………………………………………………………………………….…….

17) Do you think that the INR is a reliable test for monitoring VKA medication ?

yes no don’t know

18) In your opinion, what should be shown on the final INR results report?

an indication of medication by VKA the recommended INR target values

the PT equivalent the result of the previous INR

the dose of VKA at the time when the sample was taken

a presentation showing the current result and some previous results in the form of a line-graph or diagram.

other:

III - Coordination with other health-care professionals

A - Transmission of information

19) Do you know the desired target values for each patient who comes to the laboratory on a regular basis for VKA monitoring?

yes, always yes, often sometimes rarely never

20) If yes, who usually gives you this information ?

the patient a member of his family his family doctor

the home-care nurse the specialist … other:………

B - Transmission of results

21) Where the INR is within the therapeutic range:

- *to whom do you usually send the result ?* ………………………………………………...….

…………………………………………………………………………………………………...

- *within what sort of time frame is the result usually transmitted ?*

< 24 hours 24 - 48 hours 48 - 72 hours > 72 hours

- *by which means do you usually send this result ?*

by Internet by fax by telephone by mail other:

22) Where the INR is outside the therapeutic range  (<1.5 or >4.5):

- *To whom do you usually send the result?……………………………………………………*

- *within what sort of time frame is the result usually transmitted ?*

< 3 hours < 12 hours < 24 hours < 48 hours > 48 hours

- *by which means do you usually send this result ?*

. by Internet by fax by telephone by mail other :

23) Who makes a call to you to get the INR result ?

the patient yes, often sometimes rarely never

the doctor yes, often sometimes rarely never

the home-care nurse yes, often sometimes rarely never

the family member yes, often sometimes rarely never

other:

C - The patient and the doctor

24) Do you ever discuss the dosage with the patient :

- *where the INR is stable ?*

yes, always yes, often yes, sometimes rarely never

- *where the INR is above the therapeutic range ?*

yes, always yes, often yes, sometimes rarely never

- *where the INR is below the therapeutic range ?*

yes, always yes, often yes, sometimes rarely never

25) If yes, do you inform the family doctor?

yes, always yes, often yes, sometimes rarely never

26) If yes, what sort of advice do you give to the patient ?

*-* where the INR is stable:………………………………………………………………………..

………………………………………………………………...…………………………………

- *where the INR is above the therapeutic range:* …..…………………………...………..…

……………………...……………………………………………………………………………

…………………...………………………………………………………………………………

- *where the INR is below the therapeutic range:* ………………………….....................….

………………………….………………………………………………………………………..

……………………….…………………………………………………………………………..

27) Do some doctors ever ask you to manage the INR yourself and adjust the VKA dosage when they are absent for a day or so?

yes, always yes, often yes, sometimes rarely never

D - « ideal health-care coordination »

28) What would be your idea of “ideal coordination” between health-care professionals involved in following up patients taking a VKA?

…………………………………………………………………………………………………...………………………………………………………………………………………….........…..………………………………………………………………………………………………...……………………………………………………………………………………………….……..……………………………………………………………………………………..........……….
